# POLICY SERIES: INTERDISCIPLINARY PUBLIC POLICY DISCUSSION SESSION

**DOI:** 10.1093/geroni/igad104.1519

**Published:** 2023-12-21

**Authors:** George Taffet, Brian Lindberg

## Abstract

This interactive session is an interdisciplinary look at the impact of social isolation and loneliness policy issues as we age. This session, organized by the GSA Public Policy Committee, will provide both GSA section leadership and attendees an opportunity to have an open dialogue on important public policy issues of significance in the field of aging. The panelists represent the six sections of GSA: ESPO, BS, BSS, SRPP, HS, and AGHE.

